# Smad6 determines BMP-regulated invasive behaviour of breast cancer cells in a zebrafish xenograft model

**DOI:** 10.1038/srep24968

**Published:** 2016-04-26

**Authors:** Miriam de Boeck, Chao Cui, Aat A Mulder, Carolina R Jost, Souichi Ikeno, Peter ten Dijke

**Affiliations:** 1 Department of Molecular Cell Biology and Cancer Gemonics Centre Netherlands, Leiden University Medical Centre, 2300 RC Leiden, The Netherlands; 2 Department of Molecular Cell Biology, Electron Microscopy Section, Leiden University Medical Center, 2300 RC Leiden, The Netherlands; 3 Laboratory of Biochemistry, Showa Pharmaceutical University, Tokyo 194-8543, Japan; 4 Ludwig Institute for Cancer Research, Science for Life Laboratory, Uppsala University, Box 595, 75124 Uppsala, Sweden

## Abstract

The transforming growth factor-β (TGF-β) family is known to play critical roles in cancer progression. While the dual role of TGF-β is well described, the function of bone morphogenetic proteins (BMPs) is unclear. In this study, we established the involvement of Smad6, a BMP-specific inhibitory Smad, in breast cancer cell invasion. We show that stable overexpression of Smad6 in breast cancer MCF10A M2 cells inhibits BMP signalling, thereby mitigating BMP6-induced suppression of mesenchymal marker expression. Using a zebrafish xenograft model, we demonstrate that overexpression of Smad6 potentiates invasion of MCF10A M2 cells and enhances the aggressiveness of breast cancer MDA-MB-231 cells *in vivo*, whereas a reversed phenotype is observed after Smad6 knockdown. Interestingly, BMP6 pre-treatment of MDA-MB-231 cells induced cluster formation at the invasive site in the zebrafish. BMP6 also stimulated cluster formation of MDA-MB-231 cells co-cultured on Human Microvascular Endothelial Cells (HMEC)-1 *in vitro*. Electron microscopy illustrated an induction of cell-cell contact by BMP6. The clinical relevance of our findings is highlighted by a correlation of high Smad6 expression with poor distant metastasis free survival in ER-negative cancer patients. Collectively, our data strongly indicates the involvement of Smad6 and BMP signalling in breast cancer cell invasion *in vivo*.

Breast cancer is one of the most prevalent malignant diseases among women. The primary tumour is generally well operable, as a result breast cancer related mortality is caused by distant metastases, not by the primary tumour. It is therefore important to gain insight into the process of breast cancer dissemination. The primary step of metastasis formation is the acquisition of motility and invasive properties by cancer cells, associated with epithelial-mesenchymal transition (EMT)^1^. Epithelial plasticity is regulated by various pathways, the transforming growth factor (TGF)-β pathway being a well-studied inducer of EMT and breast cancer invasion and metastasis^2^. The role in breast cancer of another branch of the TGF-β family, namely the bone morphogenetic proteins (BMPs), is less well understood.

BMPs activate the pathway by binding to BMP type I and type II receptors, inducing the formation of a heteromeric receptor complex, which can phosphorylate and thereby activate downstream signalling molecules called Smads^3^. The BMP receptor-regulated Smad1, 5 and 8 can form complexes with common mediator Smad4. Active Smad complexes translocate to the nucleus where they regulate the transcription of target genes. BMP signalling is intricately controlled by extra- and intracellular regulators. Negative feedback is achieved by the direct upregulation of inhibitory Smad6^4^ and 7^5^.

BMPs are versatile signalling molecules, known to regulate processes such as cell proliferation, differentiation and motility. The BMP pathway is crucial for embryonic development and adult tissue homeostasis and perturbation of the pathway has been associated with for instance cardiovascular and musculoskeletal diseases^6^,^7^. Due to their ubiquitous expression and potent effects on cell behaviour, the role of BMPs in the development and progression of cancer is an intriguing topic.

BMP pathway components have been found to be misexpressed in breast cancers^8^,^9^,^10^,^11^,^12^ and BMP stimulation or inhibition affects breast cancer cell behaviour^13^,^14^,^15^,^16^,^17^,^18^,^19^,^20^,^21^,^22^. However, the reported effects vary between different BMPs and are highly context dependent. BMP6 was shown to be downregulated in breast cancers^23^. Moreover, hyper-methylation of the BMP6 promoter and consequent low expression of BMP6 was found specifically in the more aggressive estrogen receptor negative (ER-) breast cancer subtype^12^. Increased expression of negative regulators of BMP signalling was found in highly metastatic cells^24^,^25^. Hence, BMP signalling is thought to have anti-metastatic activity. There are however also conflicting reports and it appears BMP may have a more dualistic role, analogous to its family member TGF-β^26^.

To study the early stages of tumour cell dissemination and metastasis we make use of a zebrafish breast cancer xenograft model. The zebrafish is an ideal vertebrate model due to features such as optical transparency, low maintenance costs, the availability of tissue specific transgenic lines and genomic conservation. The embryonic zebrafish model is widely used as a powerful platform for xenotransplantation of human tumour cells and non-invasive imaging^27^,^28^. In the present study we investigated the role of BMP signalling in early metastatic processes. By performing Duct of Cuvier implantation of breast cancer cells in zebrafish embryos, we can monitor the effect of manipulating BMP signalling on invasion and micro-metastasis. We inhibited BMP signalling specifically in the human breast cancer cell lines through stable overexpression of inhibitory Smad6 and found that Smad6, and thus the inhibition of BMP signalling, significantly enhances breast cancer cell invasion *in vivo.*

## Results

### Flag-Smad6 overexpression potentiates MCF10A M2 invasive behaviour

**Figure 1:**
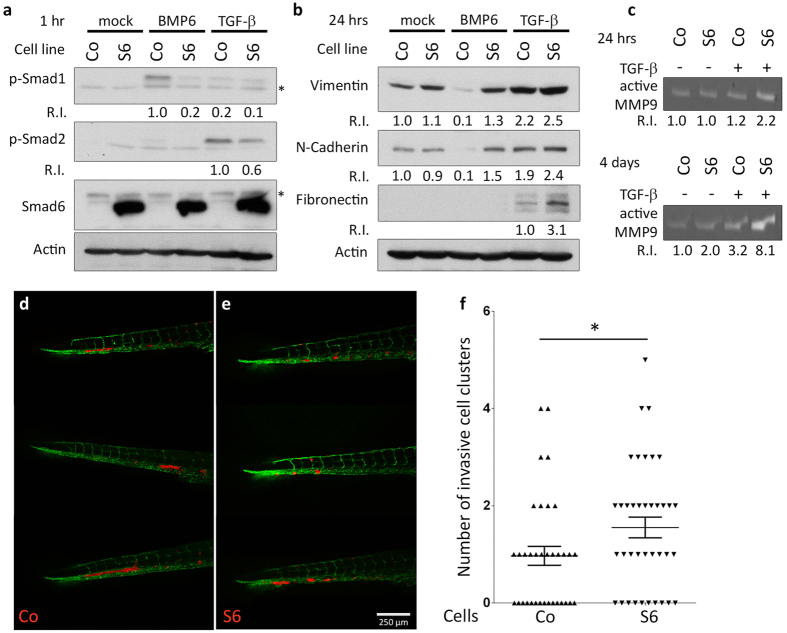
Overexpression of Smad6 in MCF10A M2 blocks BMP signalling and potentiates invasion. (**a,b**) Western blot analysis of total protein from control (CO) and Flag-Smad6 stable expression (S6) MCF10A M2 cell lines treated with 50 ng/ml BMP6 or 0.5 ng/ml TGF-β for 1 hr (**a**, *aspecific bands) and 50 ng/ml BMP6 or 5 ng/ml TGF-β for 24 hrs (**b**). (**c**) Gelatin zymogram showing MMP9 activity in conditioned medium of mock and TGF-β treated MCF10A M2 cells. (**d,e**) Representative images of 6 dpi zebrafish larvae showing the invasion of control (CO) (**d**) and Smad6 overexpression (S6) (**e**) MCF10A M2 cells. (**f**) Quantification of invasive cluster numbers in CO and S6 MCF10A M2 injected zebrafish larvae. R.I.: Quantification of relative intensity. * 0.01 < p < 0.05. Scale bar: 250 μm.

The pre-malignant Ha-Ras-transformed MCF10A M2 (MCF10AT1k.cl2) breast epithelial cell line with stable mCherry expression was used to elucidate the effects of Smad6 overexpression on epithelial plasticity and invasion. Stable Flag-Smad6 or empty vector control cell lines were established using lentiviral transduction. Stable Flag-Smad6 overexpression showed inhibition on BMP6-induced phosphorylation of Smad1, but only minor attenuation on TGF-β-induced phosphorylation of Smad2, illustrating specific blocking of BMP signalling in these cells (Fig. 1a). MCF10A M2 cells have epithelial characteristics, but are responsive to TGF-β-induced EMT marker expression. Basal expression of the mesenchymal markers Vimentin and N-Cadherin was suppressed by BMP6 stimulation and this was blocked by Flag-Smad6 overexpression. Moreover, TGF-β-induced expression of Fibronectin and MMP9 was enhanced by Flag-Smad6 overexpression (Fig. 1b,c). These results indicated that overexpression of Smad6 could potentially alter the balance of TGF-β and BMP signalling in breast cancer cells, which may change their invasive properties by elevating the expression of TGF-β responding genes.

MCF10A M2 cells can be used to study cell invasion in a zebrafish xenograft model^28^. This cell line has an intermediate invasive capacity and shows a clustered invasion phenotype when injected in the circulation of zebrafish embryos. Where empty vector control MCF10A M2 cells show mostly only one invasive cluster at the caudal haematopoietic tissue (CHT) region of the zebrafish embryo, Flag-Smad6 overexpression induced multiple invasive clusters of cells. Representative images of MCF10A M2 cell clusters at the CHT region at 6 days post-implantation (dpi) are shown in Fig. 1d,e. Quantification of the number of invasive cell clusters shows a significant increase elicited by Smad6 (Fig. 1f). This indicates Flag-Smad6 overexpression mediated inhibition of BMP signalling enhanced invasiveness of MCF10A M2 cells *in vivo*.

### MDA-MB-231 cell invasion is stimulated by Smad6

**Figure 2:**
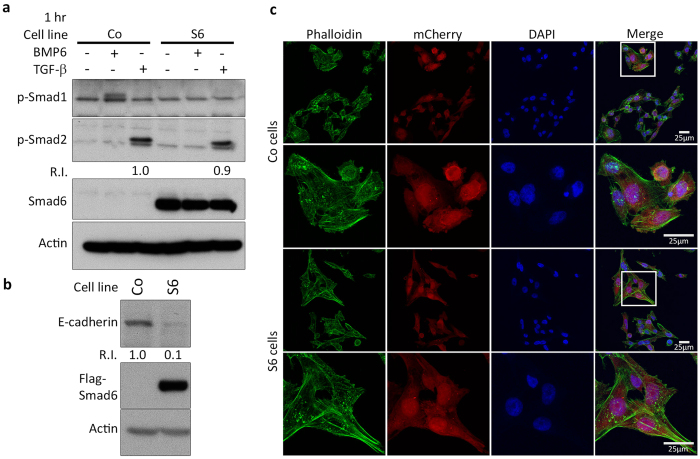
Smad6 overexpression changes MDA-MB-231 cell morphology. (**a,b**) Western blot analysis of total protein from control and Flag-Smad6 stable expression MDA-MB-231 cell line treated with 50 ng/ml BMP6 or 1 ng/ml TGF-β. (**c**) Fluorescent staining analysis of CO (top 2 panels) and S6 (bottom 2 panels, squared areas are enlarged in the lower panels) MDA-MB-231 cells to detect Alexa Fluor 488 Phalloidin-stained F-actin filaments. R.I.: Quantification of relative intensity. Scale bar: 25 μm.

MDA-MB-231 is a highly metastatic breast cancer cell line with a mesenchymal phenotype and has been widely used in xenograft models. Stable overexpression of Flag-Smad6 in MDA-MB-231 cells inhibited BMP signalling, but not TGF-β signalling (Fig. 2a). The already low basal expression of E-Cadherin in these cells was further reduced in the Flag-Smad6 expressing cells (Fig. 2b). Furthermore, a change in cell shape was observed during culture of the stable cell lines. Under normal growth medium conditions, Flag-Smad6 overexpressing cells are more elongated and spindle-shaped compared to empty vector control cells. This change in cell shape was observed by fluorescence imaging of the stable mCherry expression combined with F-Actin staining (Fig. 2c).

**Figure 3:**
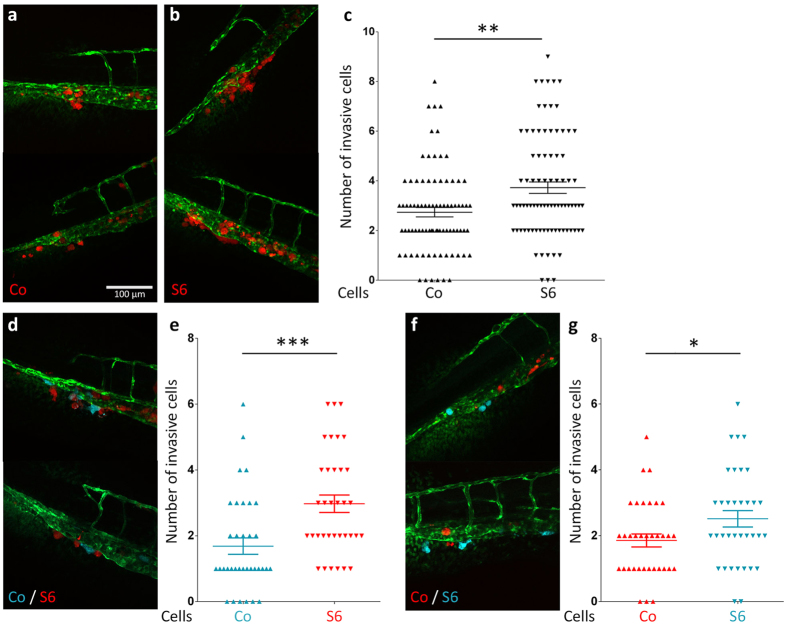
Stimulation of MDA-MB-231 cell invasion by Smad6. (**a**,**b**) Representative images of 6 dpi zebrafish larvae showing the invasion of CO (**a**) and S6 (**b**) MDA-MB-231 cells. (**c**) Quantification of invasive cell numbers in CO and S6 MDA-MB-231 injected zebrafish larvae. (**d,f**) Representative images of 6 dpi zebrafish larvae injected with pre-mixed mTurquoise-CO/mCherry-S6 cells or mTurquoise-S6/mCherry-CO cells. (**e,g**) Quantification of colour labelled CO and S6 invasive cell numbers in individual zebrafish larvae. Scale bar: 100 μm. *0.01 < P < 0.05; **0.001 < P < 0.01; ***P < 0.001.

Injection of MDA-MB-231 mCherry cells in the duct of Cuvier of zebrafish embryos at 48 hours post fertilization (hpf) gives rise to single-cell invasion into the tail fin^28^. Invasion can be quantified by counting the number of cells that have extravasated and invaded the tail fin tissue at 6dpi. Empty vector control and Flag-Smad6 overexpressing MDA-MB-231 cells were implanted and analysis at 6 dpi showed a significant increase in invasive cell numbers in the Flag-Smad6 overexpressing group (Fig. 3a–c). To investigate whether the overexpression of Flag-Smad6 purely has a cell autonomous effect, we employed a sophisticated double-colour experimental setup. Both mCherry and mTurquoise MDA-MB-231 cells were stably transduced with either empty vector or Flag-Smad6, after which mTurquoise-Control and mCherry-Smad6 cells (or *vice versa*) were mixed prior to implantation. Invasion was quantified by counting the mCherry and mTurquoise positive invasive cells in the same zebrafish at 6dpi. Both mCherry-Smad6 (Fig. 3d,e) and mTurquoise-Smad6 (Fig. 3f,g) cells were present in the tail fin in higher numbers than the opposite-colour control cells. This clearly indicates Smad6 overexpression stimulates MDA-MB-231 single cell invasion in a cell-autonomous manner.

### Smad6 knockdown inhibits MDA-MB-231 cell invasion in zebrafish

**Figure 4:**
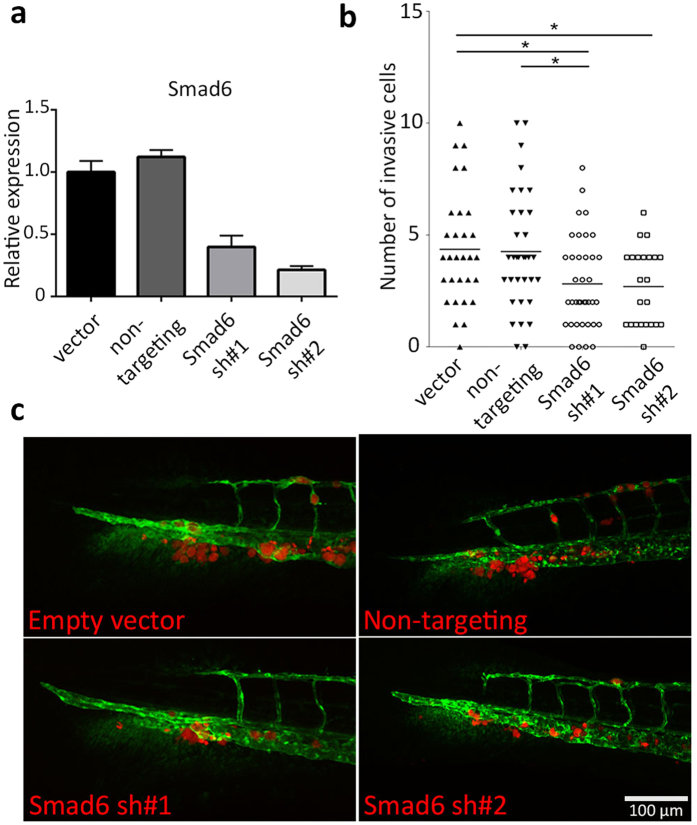
Smad6 knockdown results in reduced cell invasion of MDA-MB-231 cells. (**a**) Quantitative PCR (qPCR) analysis of Smad6 mRNA expression in MDA-MB-231 cells infected with control vector, non-targeting shRNA and two Smad6 shRNA constructs, respectively. (**b**) Quantification of invasive cell numbers in vector control, non-targeting control and Smad6 knockdown MDA-MB-231 cells injected zebrafish larvae. (**c**) Representative images of 6 dpi zebrafish larvae showing the Smad6 knockdown effect in MDA-MB-231 cell invasion. Scale bar: μm. 100 *0.01 < P < 0.05.

To further investigate the involvement of Smad6 in breast cancer cell invasion, we made use of multiple shRNA constructs to knockdown Smad6 in MDA-MB-231 mCherry cells. Smad6 knockdown was verified using qPCR (Fig. 4a). In zebrafish xenograft assays, reduced invasion was observed in Smad6 knockdown groups as compared to empty vector and non-targeting shRNA control groups (Fig. 4b–f). This result confirms the stimulatory role of Smad6 for breast cancer cell invasion in our zebrafish model.

### BMP6 pre-treatment inhibits single-cell metastasis and induces clustering of MDA-MB-231 cells in zebrafish

**Figure 5:**
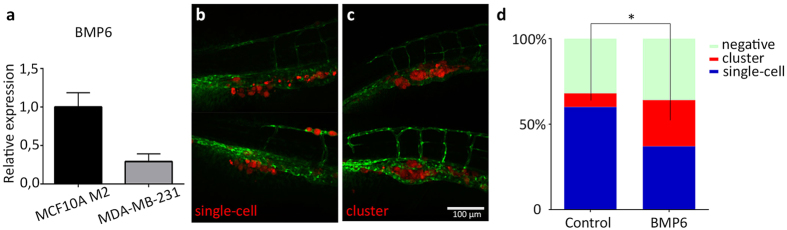
BMP6-induced cluster phenotype in MDA-MB-231 cell invasion. (**a**) qPCR analysis showing the BMP6 mRNA expression in MCF10A M2 and MDA-MB-231 cells. (**b**,**c**) Representative images of typical ‘single-cell’ invasion (**b**) and ‘cluster’ invasion (**c**) phenotype after BMP6 pre-treatment. (**d**) representation of the percentage of negative, cluster and single cell invasion phenotypes in zebrafish larvae injected with control and BMP6 pre-treated MDA-MB-231 cells. Scale bar: 100 μm. *0.01 < P < 0.05.

Since Smad6 overexpression inhibits BMP signalling and stimulates invasion in our breast cancer cell lines, we wanted to investigate the effect of BMP stimulation on cell invasion in our zebrafish model. Several studies have reported BMP6 as a potential negative regulator of breast cancer progression. BMP6 expression is downregulated in aggressive ER- breast cancers. Both MCF10A M2 and MDA-MB-231 cells are ER- cell lines, however MDA-MB-231 is generally more metastatic. We compared the expression of BMP6 in these cell lines by qPCR and found BMP6 expression is much lower in the more aggressive MDA-MB-231 cells (Fig. 5a). To study the effect of BMP6 on *in vivo* invasion of MDA-MB-231 cells, we stimulated the cells for 24 hours prior to implantation in the zebrafish. Although the difference in the total percentage of fish positive for invasion was minimal, the manner in which BMP6 pre-treated MDA-MB-231 cells invaded was different from the mock treated cells. Where mock treated cells show aggressive single-cell invasion into the tail fin, BMP6 pre-treated cells often formed tight clusters of cells in between the fish blood vessels (Fig. 5b–d). This clustered phenotype of BMP6 pre-treated MDA-MB-231 cells resembles the way the less aggressive MCF10A M2 cells behave in our zebrafish assay. BMP6 therefore changes the phenotype of aggressive MDA-MB-231 cells towards a less aggressive clustered invasion phenotype.

### BMP6 treatment of MDA MB 231 cells cultured on HMEC-1 cells induces cluster formation *in vitro*

**Figure 6:**
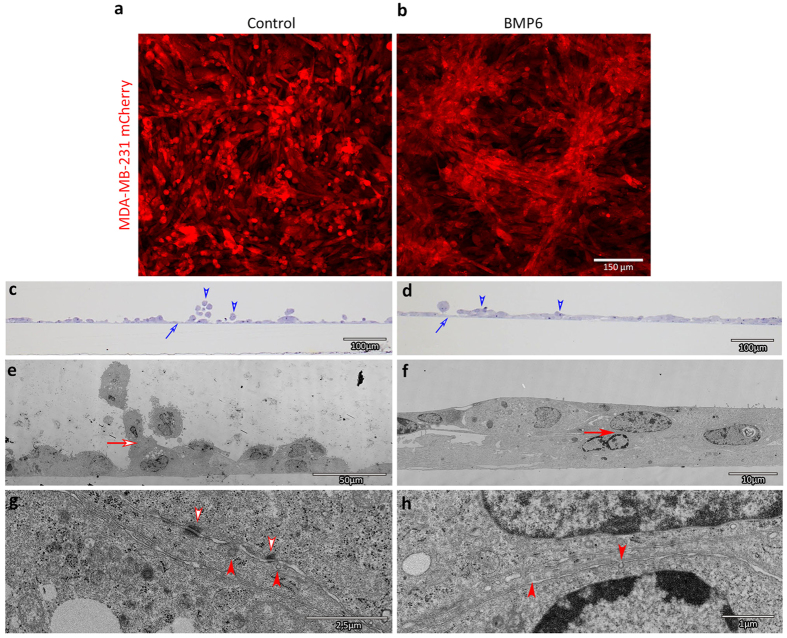
BMP6 treatment of MDA-MB-231 cells cultured on HMEC-1 cells induces multi-layered cluster formation *in vitro*. (**a,b**) Representative fluorescent images of control (**a**) and BMP6 (**b**) treated mCherry MDA-MB-231 cells seeded on a monolayer of HMEC-1 cells after 6 days. (**c,d**) Representative light microscopy images of cross sections showing the fixed MDA-MB-231 cells on the HMEC-1 monolayer. Arrow: monolayer of HMEC-1 cells. Arrow head: MDA-MB-231 cells (**e**–**h**) Electron microscopy images showing cell-cell contacts in control (**e,g**) and BMP6 treated (**f,h**) groups. Arrow: enlarged areas in the lower panels. Empty arrowhead: desmosomes between MDA-MB-231 cells. Arrowhead: membrane invaginations.

Unlike MCF10A M2 cells, MDA-MB-231 cells do not make many cell-cell contacts *in vitro* when grown in a subconfluent monolayer. Treatment of the cells with BMP6 does not change this phenotype. However, in the zebrafish we observed BMP6 pre-treated MDA-MB-231 cells clustering in between the fish blood vessels, therefore we examined how MDA-MB-231 cells behave when cultured on top of a confluent layer of Human Microvascular Endothelial Cells (HMEC-1). Without stimulation, MDA-MB-231 cells attach loosely to the HMECs and to each other (Fig. 6a). When the co-culture was treated with BMP6, MDA-MB-231 cells not only adhered better to the HMECs, but the breast cancer cells also formed tightly packed areas where multiple cells are stacked on top of each other (Fig. 6b) This co-culture phenotype mimics the clusters formed *in vivo* by BMP6-treated cells.

To study the cell-cell attachments in our co-culture system more closely, we performed electron microscopy (EM) on cross-sections of our cultures. Cross-sections of mock-treated co-cultures under light microscopy already show the loose attachment of MDA-MB-231 cells, whereas MDA-MB-231 cells in BMP-treated co-cultures are forming multiple layers of flat cells on top of the monolayer of HMEC-1 cells (Fig. 6c,d). Electron microscopic analysis revealed mock-treated MDA-MB-231 cells only use a small membrane surface area to make cell-cell contacts partly sustained by desmosomes (Fig. 6e–g). In BMP6-treated co-cultures MDA-MB-231 cells not only adhere to HMEC-1 cells, but also to each other. BMP6 treatment of co-cultures thus enlarges the cell-cell contact surface area of MDA-MB-231 cells. In both cultures the cell-cell contact surface area show signs of active membrane recycling, as illustrated by the presence of membrane invaginations resulting from either endo- or exocytosis (Fig. 6c–f).

### High Smad6 expression is correlated with increased risk of metastasis in ER negative breast cancer

**Figure 7:**
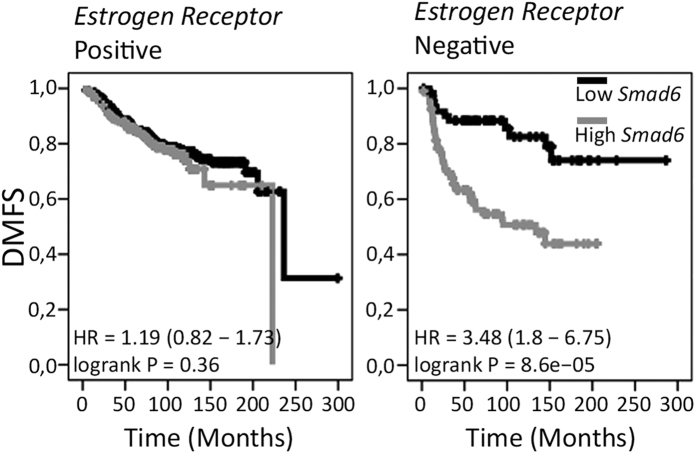
*Smad6* mRNA expression is correlated with Distant Metastasis Free Survival (DMFS) in estrogen receptor negative (ER-) breast cancers. Kaplan-Meier analysis (log-rank test) showing the correlation between high Smad6 expression and DMFS in breast cancer patients in the publicly available KM plotter database.

Our data signifies a role of BMP signalling and its inhibition by Smad6 in early metastatic processes. We used the publicly available Kaplan-Meier Plotter database to explore the clinical significance of our *in vitro* and *in vivo* findings. In this large dataset of human breast cancers^29^ we found a clear correlation of high Smad6 expression with poor Distant Metastasis Free Survival (DMFS). Interestingly, Smad6 and DMFS are only inversely correlated in estrogen receptor negative (ER-) breast cancers (Fig. 7a,b). Since ER- breast cancer is generally more aggressive and more difficult to treat, a correlation between Smad6 expression and DMFS specifically in this subset of patients clearly demonstrates the clinical relevance of Smad6 and BMP signalling in metastasis formation in breast cancer patients.

## Discussion

BMPs have been associated with breast cancer development and progression, however there are discrepancies between studies and the exact role of BMP signalling during various stages of cancer progression is still unclear. In the present study, we have found that BMP signalling and its inhibition by Smad6 are important regulators of early metastatic processes.

The clinical relevance of our findings is highlighted by the observed correlation between Smad6 expression and distant metastasis free survival specifically in ER- breast cancer patients. This striking difference between ER+ and ER- breast cancer is in line with previous findings on BMP6 expression. BMP6 was shown to be downregulated during breast cancer progression, associated with breast cancer grade and its promoter is methylated in ER- breast cancers^12^,^23^,^30^,^31^,^32^. Low BMP6 expression showed correlation with the risk of Relapse Free Survival in breast cancer patients. BMP6 has also been reported to inhibit breast cancer cell proliferation and EMT^30^,^31^,^33^,^34^. In our study, we have made use of two ER- cell lines and shown the importance of BMP signalling in EMT and for *in vivo* invasion.

Perturbations in BMP signalling have been implicated in tumorigenesis, various ligands and other signalling components are misexpressed in breast cancers^8^,^9^,^10^,^11^,^12^. Some BMP inhibitors have been shown to contribute to cancer progression and metastasis formation^24^,^25^,^35^. Since distinct BMP ligands have been described to influence breast cancer progression differentially, we decided to study the role of BMP signalling by manipulating the expression level of its inhibitory Smad. BMP signalling could be efficiently blocked by Smad6 overexpression in the ER- breast cancer cell lines that we employed. In addition, we do not exclude the possibility that Smad6 has effects independent of antagonizing BMP/Smad signalling. We detected potentiation of TGF-β-induced expression of EMT markers in FLAG-Smad6 expressing MCF10A M2 cells, indicating that the invasive properties of MCF10A M2 cells are enhanced by Smad6. Moreover, a spindle-like morphological phenotype was observed in cultured FLAG-Smad6 MDA-MB-231 cells. Phalloidin staining showed clear effects on F-actin orientation, which suggests the involvement of BMP signalling in cytoskeleton remodelling and cell adhesion in MDA-MB-231 cells.

It has been reported that Smad6 overexpression promotes lung metastasis formation of 4TO7 cells in mice^25^. However, studies further addressing the function of Smad6 in breast cancer progression *in vivo* are currently lacking. This study emphasizes the value of the zebrafish xenograft model for understanding early metastatic processes. This model has previously been used to show the importance of TGF-β signalling in MDA-MB-231 invasion^28^. Moreover, it can be easily employed to study the effects of perturbations in TGF-β signalling on breast cancer cell invasiveness. We found that injection of FLAG-Smad6 MDA-MB-231 cells in zebrafish larvae led to increased number of invasive cells at the tail fin, whereas the knockdown of Smad6 in MDA-MB-231 cells resulted in inhibition of single cell invasion. Previously, our lab has shown that MCF10A M2 cells could form clusters at the CHT region in zebrafish^28^. Interestingly, we observed that after Smad6 overexpression MCF10A M2 cells were scattered into smaller clusters around the CHT region. Collectively, this data would suggest that Smad6 has a role in cell-cell contact and communication, which affects tumour cell invasion.

Importantly, this study shows an additional benefit of the zebrafish xenograft model by clearly showing changes in the mode of invasion of MDA-MB-231 cells when pre-treated with BMP6. The phenotype of clustered MDA-MB-231 cells in between the blood vessels of the zebrafish indicates a change in cell-cell attachment induced by a short pulse of BMP activation. The observed interactions between the zebrafish endothelium and the human MDA-MB-231 cells led us to investigate this effect of BMP6 stimulation further in an *in vitro* co-culture system. By studying the cell-cell attachment of MDA-MB-231 cells in the presence of HMEC-1 cells in electron microscopy, we discovered a flattening of the cells and multi-layered cluster formation by BMP6. This interesting reaction to BMP activation in breast cancer cells could not be studied in simple monolayer culture, nor could its relevance be uncovered without the use of our zebrafish model. However, further study into the mechanism behind this BMP-induced phenotypic change is required.

In summary, we demonstrated the importance of BMP signalling as an anti-metastatic signal in ER- breast cancer cells. The inhibition of BMP signalling by the overexpression of its intracellular inhibitor Smad6 potentiated invasion in a zebrafish xenograft model. We also found that treatment of aggressive MDA-MB-231 cells with BMP6 resulted in increased cell attachment in co-culture with endothelial cells and aggregation of breast cancer cells near the zebrafish circulation at the expense of single cell invasion. Hence, BMP signalling could potentially be exploited in therapeutic intervention to prevent the metastatic spread of breast cancer.

## Methods

### Cell culture and reagents

The MCF10A-derived breast epithelial cell line M2^36^,^37^ was maintained in DMEM/F12 (Gibco, Invitrogen, Blijswijk, Netherlands) with 5% horse serum, 20 ng/ml epidermal growth factor (Upstate Biotechnology Inc, Lake Placid, NY), 10 μg/ml insulin (Sigma Chemical, St Louis, MO), 100 ng/ml cholera enterotoxin (Calbiochem, La Jolla, CA), 0.5μg/ml hydrocortisone (Sigma Chemical) and 100 U/ml Penicillin/Streptomycin (Gibco) as previously described (Steven *et al.*, 2001). Human cell lines HEK 293T and MDA-MB-231^38^ were cultured at 37 °C in DMEM-high glucose containing L-glutamine, 10% FCS and 100 U/ml Penicillin/Streptomycin (Gibco). HMEC-1 cells were cultured in MCDB 131 with 10% FCS, 10 ng/ml epidermal growth factor, 1 μg/ml hydrocortisone, Glutamax (Gibco) and 100 U/ml Penicillin/Streptomycin in 0.1% gelatin coated dishes and were used between passage 19–25. Co-culture of HMEC-1 and MDA-MB-231 was established by firstly growing HMEC-1 to confluency in HMEC-1 growth medium after which medium was removed and MDA-MB-231 cells were seeded on top at a density of 5 × 10^5^ cells per ml in MDA-MB-231 growth medium with or without addition of 250 ng/ml BMP6. Co-cultures were fixed for imaging after 5 days.

### Lentiviral transduction

Lentivirus was produced in HEK 293T cells by co-transfection of PLKO.1 constructs (empty PLKO.1, SHC007, TRCN0000019336 or TRCN0000235134) or pLV (pLV-bc-CMV-puro empty or N-terminal Flag tagged Smad6 constructs^39^ together with helper plasmids pCMV-VSVG, pMDLg-RRE (gag/pol) and pRSV-REV. Supernatants were harvested, filtered and stored at −80 °C until use. Physical particle titers were determined by p24 ELISA. Cells were transduced at 50% confluency with titer-normalized quantities of lentivirus in the presence of 5 ng/mL polybrene (Sigma, Zwijndrecht, Netherlands). After incubation for 24 hrs, virus was washed off and exchanged for growth medium containing 1 μg/ml puromycin. Stable cell lines were maintained in puromycin-containing medium for at least one week. Smad6 knockdown MDA-MB-231 cells and the respective control cells were used for analysis and injection 48 hrs after transduction.

### Microscopic analysis of cell cultures

MDA-MB-231 control or Flag-Smad6 cells were grown on coverslips. Cells were fixed in 4% PFA for 15 minutes and permeabilized in 0.1% Triton X-100 for 10 minutes. After blocking in 5% BSA, cells were incubated with Alexa Fluor 488 Phalloidin (Invitrogen) diluted 1: 200 in PBS with 0.5% BSA for 30 minutes. After washing, the coverslips were mounted using ProlongGold with DAPI (ThermoFisher Scientific). Images were taken using a Leica SP6 confocal microscope (Leica, Rijswijk, Netherlands).

### RNA isolation and real-time quantitative PCR

Total RNA was extracted with the NucleoSpin RNA II kit (BIOKE, Leiden, Netherlands) according to the supplier’s manual. cDNA was synthesized with the RevertAid First Strand cDNA Synthesis Kit (Thermo Scientific, Leusden, Netherlands). Real-time quantitative PCR (RT-qPCR) was performed on a CFX connect real-time PCR system (Bio-Rad, Veenendaal, Netherlands). All data were analyzed in triplicate and normalized to ARP. The primer sequences used for PCR were as follows: Smad6, 5′-ACAAGCCACTGGATCTGTCC-3′ and 5′-ACATGCTGGCGTCTGAGAA-3′, BMP6, 5′-TGCAGGAAGCATGAGCTG-3′and 5′-GTGCGTTGAGTGGGAAGG-3′, ARP, 5′-CACCATTGAAATCCTGAGTGATGT-3′ and 5′-TGACCAGCCGAAAGGAGAAG-3′.

### Western blot analysis

Cells were lysed in 300 μl lysis buffer (50 mM Tris-HCl, pH 7.4, 150 mM NaCl, 0,5% Triton X-100, protease inhibitor cocktail (cOmplete tablets, Roche)) for 10 min at 4 °C. After 10 min centrifugation at 13200 rpm at 4 °C, protein concentration in the supernatant was measured using the BCA protein assay kit (ThermoFisher). Equal amounts of protein were used for immunoblotting, using the Bio-Rad mini-gel running system. Antibodies used in this study were: β-actin (A5441, Sigma), Flag (F3165, Sigma), SMAD6 (sc-13048, Santa Cruz Biotechnology), rabbit polyclonal anti-phosphorylated Smad2 or anti-phosphorylated Smad1/5/8 (Ludwig Institute for Cancer Research, Uppsala, Sweden), E-Cadherin (610181, BD).

### Zebrafish maintenance

This study was approved by The Institutional Committee for Animal Welfare of the Leiden University Medical Center (LUMC). Zebrafish and embryos were maintained according to standard procedures. The transgenic fish line Tg(fli1:GFP) was used in this study as described before^27^,^40^. All experiments were performed in accordance with approved guidelines and regulations.

### Embryo preparation and tumor cell implantation

Tg(Fli1:GFP) zebrafish embryos were dechorionated at 2 days post fertilization (dpf). Single cell suspensions of MDA-MB-231 or MCF10A M2 cells were re-suspended in PBS and kept at 4 °C before injection. Cell suspensions were loaded into borosilicate glass capillary needles (1 mm O.D. × 0.78 mm I.D.; Harvard Apparatus). Injections were performed with a Pneumatic Picopump and a manipulator (WPI, Stevenage, UK). Dechorionated embryos were anaesthetized with 0.003% tricaine (Sigma) and mounted on 10-cm Petri dishes coated with 1% agarose. Approximately 400 cells were injected at the duct of Cuvier (DOC). Injected zebrafish embryos were maintained at 33 °C. All the experiments were repeated at least three times with at least 30 embryos per group.

### Microscopy and analysis

Zebrafish were fixed with 4% paraformaldehyde at 4 °C overnight. Fixed embryos were imaged in PBS with 0.1% Tween-20 (Merck, Amsterdam, Netherlands) with a Leica SP5 STED confocal microscope (Leica). Confocal stacks were processed for maximum intensity projections with Image J. Brightness and contrast of images were adjusted with Adobe Photoshop CS6.

### Electron Microscopy

Cell co-cultures were fixed in 1.5% glutaraldehyde, post-fixed in 1% osmium tetroxide. After dehydration up to ethanol 100% in the culture dishes, the cells were embedded in epoxy resin LX-112 (Ladd research). After polymerization the embedded cells were taken out of the dish and re-embedded in an embedding mold to enable transversal sectioning. Semithin sections (1 μm) stained with a toluidine blue solution (1% in AD) were examined with Zeiss axioplan 2 imaging microscope system. Ultrathin sections (80nm) were made and post stained with uranyl acetate and lead citrate. Electron microscopy images were obtained in a FEI Tecnai Twin (camera: FEi, Eagle CCD camera) or a FEI Tecnai F20Twin (camera: US4000Gatan) both operating at 120kV. A total of 1350 at binning 1 (Fig. 6e–g) or 2263 at binning 2 (Fig. 6f–h) overlapping images were collected and stitched together into separate images as previously described^41^. In the resulting datasets cell-cell contact areas between cells growing on the feeder layer were examined and representative images are shown in Fig. 6g,h.

### Statistical analysis

Statistical analysis was performed using Prism 4 software (GraphPad La Jolla, USA). Results are expressed as the mean ± SEM. Student’s t-test or one-way analysis of variance (ANOVA) were performed followed by the Tukey’s method for multiple comparison. P < 0.05 was considered to be statistically significant (*0.01 < P < 0.05; **0.001 < P < 0.01; ***P < 0.001).

## Additional Information

**How to cite this article**: de Boeck, M. *et al.* Smad6 determines BMP-regulated invasive behaviour of breast cancer cells in a zebrafish xenograft model. *Sci. Rep.* **6**, 24968; doi: 10.1038/srep24968 (2016).
